# Clinical utility and psychometric validity of the International Adjustment Disorder Questionnaire in a sample of South African working adults

**DOI:** 10.4102/sajpsychiatry.v32i0.2578

**Published:** 2026-04-22

**Authors:** Charles H. van Wijk, Chris J.B. Muller, Blanche N. Andrews

**Affiliations:** 1 Division of Health Systems and Public Health, Department of Global Health, Faculty of Medicine and Health Sciences, Stellenbosch University, Tygerberg, Cape Town, South Africa; 2 Department of Statistics and Actuarial Science, Faculty of Economic and Management Science, Stellenbosch University, Stellenbosch, South Africa

**Keywords:** adjustment disorder, clinical utility, IADQ, ICD-11, occupational health, resilience, screening, validation

## Abstract

**Background:**

Adjustment disorder (AjD) is a significant yet often underdiagnosed condition in workplace mental health. The International Adjustment Disorder Questionnaire (IADQ) has shown promise as a screening tool, but its clinical utility within South African (SA) occupational settings remains unexplored.

**Aim:**

To assess the psychometric validity and clinical utility of the IADQ in an educated SA workplace population.

**Setting:**

The research was conducted across various occupational sectors in South Africa.

**Methods:**

A cross-sectional survey included the IADQ alongside validated measures of depression, anxiety, post-traumatic stress disorder and psychological resilience. Confirmatory factor analysis assessed structural validity, while performance of the IADQ algorithm was evaluated against clinical interviews. A grey zone approach was considered to refine screening thresholds.

**Results:**

Adjustment disorder point prevalence was 4.3% in this sample. Confirmatory factor analysis supported the two-factor model of AjD symptoms, with measurement invariance observed across gender and language groups. The IADQ algorithm demonstrated good diagnostic accuracy for screening purposes, with sensitivity and specificity values exceeding 0.95. Psychological resilience was associated with lower odds of AjD.

**Conclusion:**

The IADQ demonstrated good psychometric properties and clinical utility for comparable workplace mental health screening. The grey zone approach may offer a practical framework for graduated triage in resource-limited settings.

**Contribution:**

Our study supported the global applicability of the two-factor AjD symptom model, provided updated AjD prevalence rates for a SA workplace sample, and considered a refined screening method for use in occupational mental health services.

## Introduction

Adjustment disorder (AjD) is a common yet frequently under-recognised condition in occupational mental health, where psychosocial stressors are prevalent. Reliable identification of AjD is essential for early intervention, prevention of chronic impairment and efficient use of limited mental health resources. Our study examined the psychometric validity and clinical utility of the International Adjustment Disorder Questionnaire (IADQ) in a South African (SA) educated working population, with particular attention to its structural validity, diagnostic usefulness and practical screening thresholds in a resource-constrained healthcare context.

### Adjustment disorders

Adjustment disorders are maladaptive reactions to identifiable, mostly non-traumatic, psychosocial stressors or multiple critical life events such as job loss, divorce, socioeconomic problems or severe disease or accidents.^1,2^ Historically, diagnosing AjD was challenging due to its vague conceptualisation. For example, the International Classification of Diseases (ICD)-10 described it as a transitional period following the onset of stressors, without specifying unique symptoms.^3^ Instead, it encompassed a broad range of emotional and behavioural symptoms that impair daily functioning.^3^ The AjD diagnosis has also been criticised for its difficulties in distinguishing AjD from normal stress reactions and other clinical and subclinical mental disorders.^4,5,6^

In response, the ICD-11 reconceptualised AjD with a focus on two core symptom clusters: ‘preoccupation with the stressor or its consequences’ and ‘failure to adapt’.^2^ These core symptoms usually manifest within a month of the stressor and result in significant impairment in personal, social, educational or professional domains, and typically resolve within 6 months, unless the stressor persists for a longer duration.^2^

For an ICD-11 diagnosis of AjD, the following criteria must be met^2^:

- An identifiable psychosocial stressor.
- At least one symptom of preoccupation with the stressor and its consequences, as well as at least one symptom reflecting difficulties in adapting to the stressor.
- Symptom onset usually within a month of the stressor.
- Significant functional impairment due to the symptoms.

The ICD-11 further specifies that AjD is only diagnosed when the symptoms described above are not better explained by another clinical disorder (such as mood disorder, post-traumatic stress disorder [PTSD] or prolonged grief disorder).

Adjustment disorder is among the most frequently encountered mental health conditions in clinical practice.^7,8^ Epidemiological studies report wide variability in AjD prevalence, among other due to the heterogeneity of outcome measures, study populations and sampling methods. General population studies suggest a 2% prevalence,^9^ but rates are notably higher in specific groups, such as the recently bereaved (18%),^10^ recently retrenched workers (27%),^11^ and patients with recurrent breast cancer (35%).^12^ South African data on AjD are sparse. A small SA Navy study with limited generalisability reported a point prevalence of 6.9% with high (> 30%) comorbidity.^13^

Several risk factors for AjD have been identified. Research on gender differences in AjD remains inconsistent. Some studies suggest higher risk among women, while others report equal distribution across gender groups.^14,15,16,17^ Younger age, unemployment status, physical injury and illness, history of mental disorders, low social support, and lower education have all been associated with an increased risk for AjD.^15,17^ Psychological resilience has been shown to mitigate AjD risk.^18^

Although AjD symptoms are typically expected to resolve within 6 months, they may become chronic or transition into more severe disorders if untreated.^19,20,21^ This points to the importance of early identification and intervention to prevent prolonged distress and functional decline. However, this poses challenges within resource-restricted settings, and here psychometric instruments may be useful.

Given that many psychosocial stressors arise in occupational contexts, where increased mental health risk has also been documented,^22^ the workplace represents an important setting for the identification of AjD. As psychosocial problems in the workplace can increase the risk of AjD,^23^ and conversely, AjD can significantly impair occupational functioning, validating a screening tool like the IADQ is highly relevant to occupational health. In SA, employers have a legal obligation to prevent work-related mental ill health, and to manage associated incapacity.^24,25^ Tools that support early identification are essential for timely intervention and support in the workplace.

### Psychometric assessment of adjustment disorders

Two questionnaires assess ICD-11 AjD symptoms: the Adjustment Disorder New Module (ADNM)^26^ and the IADQ.^16^ The ADNM predates the finalised ICD-11 criteria and deviates somewhat from its narrative description. The IADQ, by contrast, aligns more closely with ICD-11 and offers an improved measure relative to the ADNM by providing a simple and clinically useful diagnostic algorithm.^27^ The IADQ comprises four sections^16^:

- The psychosocial stressor checklist includes eight specific life stressors and one open-ended item. Responses are dichotomous (No = 0, Yes = 1). If one or more stressors are endorsed, the participants are eligible to proceed to the next section.
- The symptom scale consists of six items, with three items each assessing preoccupation and failure to adapt. Responses are rated on a 5-point scale (0 = *not at all* to 4 = *extremely*) with participants asked to indicate ‘… how much you have been bothered by each of the following problems in the past month’.
- A binary question confirming symptom onset within a month of the stressor.
- The functionality section consists of three items measuring impairment within the social domain, at work or education, or within other important aspects of life, also rated on a 5-point scale (0 = *not at all* to 4 = *extremely*).

The IADQ has demonstrated good psychometric properties across various populations and translations within Western, educated, industrialised, rich and democratic (WEIRD) countries^14,16,28,29,30^: Confirmatory factor analyses (CFA) support both one-factor and two-factor models, with the two-factor model showing a superior fit. Excellent internal consistency has been reported, and IADQ scores showed strong correlations with measures of depression, anxiety, acute stress and negative emotions (with the highest correlations observed for the ‘failure to adapt’ factor),^30^ and more modest correlations with PTSD, complex PTSD and positive emotions. No attempts to validate the IADQ in African contexts could be located in preparation of our study.

### Current study

In spite of internationally demonstrated evidence of validity, the IADQ cannot unconditionally be used in SA. South Africa has a resource-constrained healthcare system,^31,32^ including mental healthcare,^33,34^ where the local healthcare ecology may not mirror that of WEIRD countries. Local stressors such as human immunodeficiency virus (HIV) status, adverse childhood experiences (ACE) and interpersonal violence may further be contextually relevant, in addition to employment status.^35,36,37,38^ Therefore, evidence of clinical utility in context is required before widespread adoption can be considered. Additionally, to ensure fairness in screening, evidence of local validity is also required in terms of SA legislation.^39^ Healthcare provision in SA is further constrained by language challenges,^40,41^ where health providers and users may come from different language backgrounds. In practice, particularly in higher educated groups, English is still used on the assumption of best common language.^42^ Our study thus aimed to investigate the clinical utility and psychometric validity of the English-version IADQ in an SA workplace population. It was both a replication of validation studies from WEIRD countries, and an extension by considering grey zone scoring as a potential tool for practical risk identification in resource-restricted settings. Additionally, it aimed to explore the clinical utility of the English-version IADQ in a multilingual, educated, workplace sample. In support of these aims, the study pursued five objectives:

- Examining the endorsement of psychosocial stressors offered by the IADQ.
- Estimating point prevalence of AjD using the IADQ diagnostic algorithm.
- Evaluating the IADQ’s psychometric properties, namely (1) structural validity though CFA and internal consistency analysis; and (2) concurrent validity by correlating IADQ scores with other clinical measures.
- Investigating socio-demographic variables and psychological resilience as risk and protective factors.
- Exploring clinical utility of the IADQ for practical application.

## Research methods and design

### Data source and participants

This cross-sectional study used medical records of full-time employees who participated in employer-sponsored routine occupational health assessments during 2023–2024. Cases were excluded when there were missing data for the socio-demographic variables of age and self-reported gender and language, or for the IADQ, other clinical scales or interview outcome. The present analysis was conducted on the first 2000 consecutively enrolled participants meeting these criteria. This sample size exceeds recommended thresholds for CFA and psychometric validation, thereby ensuring stable parameter estimation and adequate statistical power for the planned analyses.

The routine occupational health assessment was mandatory, while the completion of the psychometry was voluntary. In practice, participants completed the battery of psychometric scales upon early morning arrival at their occupational health assessment, and partook in an interview with a psychologist in the afternoon, or the next day.

Participants ranged in age from 20 to 60 (*M* = 35.9, standard deviation [s.d.] = 8.4) and included 604 women (30.2%) and 1396 men (69.8%). English was the first language for 375 individuals (18.8%), while the remaining 1625 (81.3%) reported one of the other 10 official SA-spoken languages as their mother tongue.

All participants had completed at least a Grade 12 education along with formal vocational training. They represented diverse vocational backgrounds, among others technical and engineering (26%), maritime (18.4%), clerical and administrative (13.5%), security (21.1%) and catering and hospitality (6.5%). All were skilled workers in full-time salaried employment; data on employment duration or socioeconomic status were not available.

### Measures

#### International adjustment disorder questionnaire

The IADQ was administered in its standard English format, available at https://doi.org/10.1111/acps.13126. A probable diagnosis of AjD was determined using the established IADQ algorithm,^16^ which requires:

- A psychosocial stressor score ≥ 1.
- Endorsement of at least one preoccupation symptom and one failure to adapt symptom (each with a score of ≥ 2).
- Symptom onset within 1 month of the stressor.
- Endorsement of at least one functional impairment criterion (with a score of ≥ 2 on any of the three functional impairment items).

A total severity score can also be calculated by summing the three preoccupation and three failure to adapt symptom scores, with a possible range of 0–24.

#### Other psychological scales

The Patient Health Questionnaire (PHQ-9) screens for symptoms of depression and has been validated in SA. It was administered in its standard 9-item English format.^43,44^ McDonald’s ω = 0.851 in this sample.

The Generalised Anxiety Disorder (GAD-7) Scale screens for symptoms of generalised anxiety and has been validated in SA. It was administered in its standard 7-item English format.^44,45,46^ McDonald’s ω = 0.875 in this sample.

The primary care PTSD for Diagnostic and Statistical Manual of Mental Disorders (DSM)-5 screener (PC-PTSD-5), is used to screen for core PTSD symptoms, and was administered in its original 0–5 scoring English format.^47^ McDonald’s ω = 0.884 in this sample.

The Connor–Davidson Resilience Scale (CD-RISC-10) assesses general psychological resilience. Responses to the 10-item English-version, validated in SA, were available for a subsample of *N* = 1600.^48,49^ McDonald’s ω = 0.897 in this sample.

#### Clinical interview

Each participant underwent a clinical interview with a psychologist as part of their occupational health assessment. Diagnosis of AjD was based on semi-structured interviews guided by the ICD-11 narrative diagnostic criteria. Interviewers were trained on the ICD-11 criteria, and received regular supervision to support diagnostic consistency; however, because interviews formed part of routine clinical care, no independent diagnostic verification was conducted. Interviewers did not have access to the IADQ responses. Diagnoses recorded in the archived dataset served as the reference standard for evaluating the performance of the IADQ algorithm.

### Data analysis

Structural analyses were conducted using R, while all other analyses were performed using IBM Corp. IBM SPSS Statistics for Windows, Version 29.0. Armonk (NY): IBM Corp.; 2022. The IADQ psychosocial stressor list was used to identify participants who had endorsed at least one life stressor; only these participants were included in subsequent analyses. Prior to conducting inferential analyses, relevant statistical assumptions were examined and found to be adequately met. Adjustments for multiple comparisons were not applied, as the analyses formed part of theory-driven psychometric validation rather than exploratory hypothesis testing. Endorsement (objective 1) was examined using frequency analysis. Descriptive statistics were then calculated for the six symptom indicators of AjD. Point prevalence (objective 2) was estimated using the established IADQ diagnostic algorithm,^16^ with 95% confidence intervals calculated for the resulting algorithm-derived classification.

Structural analyses (objective 3a) were conducted in R version 4.4.2,^50^ where CFA models were fitted using the lavaan package (v06-1), and McDonald’s ω (categorical ω) was calculated using the Methods for the Behavioral, Educational, and Social Sciences (MBESS) package (v4.9.3). Dimensionality was examined through CFA to evaluate the fit of hypothesised measurement models. Following previous validation studies, two models were tested:

- A single latent factor model where all six AjD symptoms loaded onto one construct.
- A two-factor model where preoccupation (3 items) and failure to adapt (3 items) were treated as separate latent constructs, and no cross-loadings were permitted.

All models were estimated using diagonally weighted least squares, with symptom variables treated as ordinal.

In CFA, ideal model fit is indicated by a small, non-significant Chi-square (χ^2^), but this is seldom achieved in samples > 400.^51^ Therefore, in larger samples, additional fit indices are typically considered: a root mean square error of approximation (RMSEA) between 0.05 and 0.08, the comparative fit index (CFI) > 0.90, the Tucker–Lewis index (TLI) > 0.95 and the standardised root mean square residual (SRMR) < 0.08 were interpreted as a reasonable approximate fit.^52,53^ Internal consistency reliability was assessed using McDonald’s ω, which provides a more appropriate reliability estimate than Cronbach’s α when factor loadings are not assumed to be equal; results ≥ 0.80 were regarded as evidence of good internal reliability.^54,55,56^

Measurement invariance is used to determine whether a psychological construct is interpreted consistently across groups (e.g. gender, language, diagnostic status).^57^ Invariance testing follows a stepwise process: configural invariance evaluates whether the same factor structure holds across groups; metric invariance tests whether item loadings are equivalent; and scalar invariance assesses whether item intercepts are comparable. Progression through these steps requires adequate model fit at each level, and invariance is supported when changes in Chi-square (χ^2^) between nested models are not statistically significant. The two-factor IADQ model was tested for invariance across gender (women vs. men), language (English vs. non-English first language) and diagnostic status (meeting or not meeting algorithm criteria for AjD).

Concurrent validity (objective 3b) was evaluated using bivariate correlations with other psychological measures (PHQ-9, GAD-7, PC-PTSD-5 and CD-RISC-10). Correlations above 0.30 were considered moderate and above 0.50 as strong. The role of socio-demographic and psychological variables (objective 4) was first examined using Chi-square analysis (for gender and language) and independent samples *t* tests (for age and resilience scores). The variables were then entered into a binomial logistic regression analysis to estimate their relative risk for AjD. Multicollinearity diagnostics were examined prior to model estimation. Gender was coded into two groups (women, men) based on participant self-identification, and language was coded as English as a first language versus non-English as a first language.

Finally, clinical utility (objective 5) was examined by calculating the sensitivity, specificity and positive and negative likelihood ratios (LR) of the IADQ algorithm-determined diagnosis using the clinical interview diagnosis as the reference standard.^58^ A receiver operator/operating characteristic (ROC) curve analysis was conducted, and Youden’s Index was used to determine the optimal severity score cut-off for diagnosing AjD.

Within resource-constrained environments, psychometric instruments are frequently used to screen large populations for further referral to mental healthcare provision. However, rigid cut-offs can be problematic, as intra-individual and situation-specific conditions can influence questionnaire responses. To address this limitation, a ‘grey zone’ approach was considered. The grey zone refers to the space between a lower threshold score that maximises sensitivity (the ‘at-risk’ threshold – interpreted as requiring closer monitoring) and an upper threshold score that maximises specificity (the ‘intervention’ threshold – interpreted as requiring action).^59,60^ The grey zone approach functions as a triage heuristic that supports graduated decision-making rather than binary classification. It was applied to the IADQ severity score to explore its practical application in clinical and occupational health settings. As thresholds are context-dependant, illustrative cut-offs were defined as follows: sensitivity of 95% for the lower threshold and a specificity of 95% for the upper threshold. This approach aligns with dimensional models of psychopathology and could support proportionate decision-making in occupational settings. It should be noted that grey zone categories are not diagnostic and cannot be used for prevalence estimation.

### Ethical considerations

Ethical clearance to conduct our study was obtained from the Stellenbosch University Health Research Ethics Committee (Ref. No. N25/05/046). Participants consented for their anonymised data to be used for research purposes. Only deidentified data were included in the study dataset.

## Results

### Stressor endorsement and point prevalence

The presence of one or more stressors was endorsed by 653 participants (32.7%), of which 86 met the IADQ algorithm criteria for AjD. This subsample differed from those who did not meet the stressor criterion, namely on age (on average 1 year older; *t* = 2.627, *p* < 0.05, Cohen’s *d* = 0.12) and gender (male = 31% vs. female = 37%, χ^2^ = 8.333, *p* < 0.05) and scored 2.5 points higher on the CD-RISC-10 (*t* = 8.781, *p* < 0.01, Cohen’s *d* = 0.90). There were no significant differences in terms of language backgrounds (χ^2^ = 1.094, *p* = 0.30). Subsequent structural and concurrent validity analyses were based on the 653 participants who reported one or more stressors (which was a prerequisite for AjD). In this group, no significant differences in AjD prevalence were found across gender (χ^2^ = 0.672, *p* = 0.41) or language (χ^2^ = 0.047, *p* = 0.83), and the combined group (*N* = 653) was therefore used for further analyses.

The most endorsed stressor domains were ‘Loved one’s health problems’ (32.3%), ‘Financial problems’ (27.9%) and ‘Relationship problems’ (25.3%). Further stressor and symptom endorsement data are provided in Table 1 and Table 2. As expected,^16^ preoccupation items had slightly higher mean scores than failure to adapt items.

**TABLE 1:** Endorsement of psychosocial stressors.

Psychosocial stressor	Group that endorsed one or more stressors (*N* = 653)	
	%	95% CI
Financial problems	27.9	-
Work/employment problems	12.0	-
Educational problems	12.6	-
Housing problems	19.4	-
Relationship problems	25.3	-
Personal health problems	24.0	-
Loved one’s health problems	32.3	-
Caregiving problems	11.3	-
Other	7.2	-
Cases of adjustment disorder, per algorithm (full sample)	4.3	3.4–5.2
Cases of adjustment disorder, per algorithm (stressor endorsed)	13.2	10.6–15.8

CI, confidence interval.

**TABLE 2:** Endorsement of symptoms.

Items	Group that endorsed one or more stressors (*N* = 653)				Group that met criteria for adjustment disorder (*N* = 86)			
	M	SD	*n*	%	M	SD	*n*	%
**Preoccupation**								
I worry a lot more since the stressful event(s).	1.08	1.12	-	-	2.71	0.93	-	-
I cannot stop thinking about the stressful event(s).	0.93	1.11	-	-	2.42	1.07	-	-
I often feel afraid about what might happen in the future since the stressful event(s).	1.01	1.21	-	-	2.69	1.02	-	-
Subscale score	3.03	3.11	-	-	7.81	2.42	-	-
**Failure to adapt**								
I find it difficult to adapt to life since the stressful event(s).	0.46	0.90	-	-	2.10	1.09	-	-
I find it difficult to relax and feel calm since the stressful event(s).	0.53	0.90	-	-	2.02	1.13	-	-
I find it difficult to achieve a state of inner peace since the stressful event(s).	0.64	1.06	-	-	2.63	1.10	-	-
Subscale score	1.62	2.62	-	-	6.65	2.67	-	-
Total AjD score (‘severity’ score)	4.64	5.33	-	-	14.47	4.57	-	-
**Symptom time criterion**								
Did these problems start within 1 month of the stressful event(s)?	-	-	177	27.1	-	-	86	100
**Functional impairment**								
Affected your relationships or social life?	0.65	1.11	-	-	2.33	1.29	-	-
Affected your ability to do your work or your studies?	0.51	1.01	-	-	1.88	1.28	-	-
Affected any other important part of your life?	0.46	0.97	-	-	1.73	1.31	-	-

AjD, adjustment disorder; M, mean; SD, standard deviation.

The 86 participants meeting the IADQ algorithm criteria for AjD presented an estimated conditional prevalence of 13.2% in the subsample endorsing one or more stressors. The total sample point prevalence was 4.3% (95% CI: 3.4–5.2). Of the 86 cases, 14 also met criteria for other mental disorders (eight for major depressive disorder, four for GAD, and one each for PTSD and alcohol use disorder).

### Structural validity

#### Dimensionality

As expected,^51^ the one-factor solution did not achieve a non-significant χ^2^, while the CFI, TLI and SRMR suggested acceptable fit (Table 3). The two-factor model (preoccupation and failure to adapt) did not achieve a non-significant χ^2^ either, but the values were not excessively high and the other fit indices exceeded the cut-points provided earlier. The two factors correlated strongly (*r* = 0.866), with high standard loadings within each factor (Preoccupation items 1–3 = 0.911; 0.921; 0.866; failure to adapt items 1–3 = 0.929; 0.939; 0.907). As detailed in Table 3, the two-factor model offered a superior fit to the data, and was thereafter subjected to measurement invariance testing.

**TABLE 3:** Goodness of fit statistics.

Model	χ^2^	*df*	*p*-value	CFI	TLI	RMSEA	RMSEA 90% CI	SRMR
One-factor	199.169	9	< 0.001	0.983	0.972	0.180	0.159–0.202	0.056
Two-factor	55.605	8	< 0.001	0.996	0.992	0.096	0.073–0.120	0.023

*df*, degrees of freedom; CFI, comparative fit index; TLI, Tucker–Lewis index; RMSEA, root mean square error of approximation; CI, confidence interval; SRMR, standardised root mean square.

#### Measurement invariance

The two-factor model showed acceptable configural, metric and scalar invariance (Δχ^2^ = 7.735, Δ*df* = 16, *p* = 0.96) for gender, as well as acceptable configural, metric and scalar invariance (Δχ^2^ = 13.471, Δ*df* = 16, *p* = 0.64) for language. The model further showed acceptable configural invariance for diagnosis, but did not achieve metric invariance (Δχ^2^ = 13.615, Δ*df* = 4, *p* = 0.01).

#### Internal consistency

The IADQ total scale showed good internal consistency (McDonald’s ω = 0.959), as did the two-factor subscales, namely preoccupation (McDonald’s ω = 0.888) and failure to adapt (McDonald’s ω = 0.899).

### Concurrent validity

The IADQ showed strong positive correlations with the measures of depression and anxiety, consistent with published studies, while the correlation with the PTSD screen was moderate and lower than published reports. The negative correlation with the measure of psychological resilience was low to moderate. Similar to previous reports, the associations were slightly stronger for failure to adapt than for preoccupation.^14,29,30^ The correlations coefficients are detailed in Table 4.

**TABLE 4:** Correlations between International Adjustment Disorder Questionnaire and other measures of mental health.

SA sample (*N* = 653)	IADQ total score	Preoccupation	Failure to adapt
Patient Health Questionnaire-9	0.593	0.532	0.577
Generalised Anxiety Disorder scale-7	0.626	0.541	0.633
Primary care PTSD screen for DSM-5	0.325	0.293	0.314
Connor–Davidson Resilience Scale-10 (*N* = 511)	−0.279	−0.225	−0.299

Note: All correlations significant at *p* < 0.001.

IADQ, International Adjustment Disorder Questionnaire; PTSD, post-traumatic stress disorder; SA, South African; DSM, Diagnostic and Statistical Manual of Mental Disorders.

### Risk and protective factors

There were no significant gender or language differences in AjD prevalence. A significant age difference was found between participants with and without probable AjD (*t* = 2.338, *p* < 0.05, Cohen’s *d* = 0.25), but the mean age difference was only about 1 year. Resilience scores (*N* = 511 available for this group) were significantly higher (*t* = 5.827, *p* < 0.01, Cohen’s *d* = 0.79, *M* diff = 4.7) among participants without AjD, and this variable was included in the binomial logistic regression to examine whether resilience predicted the likelihood of meeting criteria for AjD. Age, gender and language were included in the logistic regression as covariates. The overall model explained a modest 12% of the variance (Nagelkerke *R*^2^ = 0.127), and the addition of the covariates did not improve the model fit (Δχ^2^ = 4.06, *p* = 0.255). After adjustment, resilience was a significant predictor of AjD (*B* = −0.12, standard error [SE] = 0.02, Wald = 29.633, *p* < 0.001, odds ratio [OR]: 0.87). Higher general psychological resilience was significantly associated with lower odds of algorithm determined AjD, representing a 13% reduction in odds per unit increase in resilience. As expected, neither age, gender nor language was statistically significant predictors.

### Clinical utility

The IADQ identified 86 cases of AjD, with 84 identified by clinical interview. Against this reference standard, the IADQ identified one false negative and three false positives, resulting in a sensitivity of 0.988, specificity of 0.995, a LR+ of 197.6, and a LR− of 0.012. The diagnostic odds ratio was 15 659, indicating very high accuracy relative to the clinical interview in this sample.

Utility of the severity score was explored using ROC curve analysis, which showed an area under the curve of 0.969 (95% CI: 0.955–0.982). The optimal cut-off using Youden’s Index was > 8.5 (sensitivity = 90.7%, specificity = 90.8%). The grey zone approach identified scores > 7 as the lower threshold (‘require closer monitoring’) and scores > 10 as the upper threshold (‘require urgent clinical attention’). In this sample, applying the lower threshold would have added two probable cases as well as 16 cases that did not meet IADQ algorithm for diagnosis, to the list for ‘further monitoring’. Applying the upper threshold would have removed 29 cases that did not meet IADQ algorithm for diagnosis, as well as nine probable cases, from the list for ‘urgent attention’.

## Discussion

The study is the first known validation of the English-version IADQ within a SA sample. It aimed to assess the clinical utility and psychometric validity of the IADQ within an SA educated workplace population. The findings largely aligned with international validation studies, demonstrating that the IADQ performed well in detecting AjD according to ICD-11 criteria within this sample. The study further extended previous research by exploring local risk and protective factors and considering a grey zone approach for practical application in resource-limited settings.

### Prevalence estimates

The estimated AjD point prevalence of 13.2% among individuals who reported psychosocial stressors was lower than in other studies from WEIRDS countries using the same IADQ diagnostic algorithm.^28,29,30^ Likewise, the 4.3% estimated population prevalence was lower than comparable national studies,^14,16^ although it was higher than the 2% suggested in the general population,^9^ and remains significant in occupational health contexts. Placing point prevalence estimates into context remains a challenge, as reported rates of AjD appear contingent on the presence of specific stressors or screening settings (e.g. healthcare vs. general population). The current study sample may partly explain the lower prevalence rate: participants were employed adults with relative income security and access to employer-supported healthcare, including mental health services, which may have introduced a healthy worker effect, potentially reducing the prevalence of AjD compared with the general population.^15,17^ Scale characteristics, for example, English administration in a multilingual sample, could also have confounded prevalence estimates. Understanding which stressors were most frequently endorsed may provide important context for interpreting these prevalence estimates.

### Endorsement of psychosocial stressors

The most endorsed psychosocial stressor was concern about a loved one’s health. The general ageing of SA society and increasing age-associated chronic disease burden in SA^61^ may point to an increasing burden of care for ageing parents or grandparents by adult children. Clinical records indicated that endorsement of ‘financial problems’ was sometimes related to expenses for family members’ healthcare, further contributing to stress. The next highest endorsement – of financial and relationship problems – mirrors previous reports.^14,16,29^

### Psychometric properties

The results from the structural and concurrent validity analyses were promising. The CFA showed acceptable fit indices, confirming the two-factor structure (preoccupation and failure to adapt) of the IADQ. This was consistent with previous validation studies,^16,30^ as well as the ICD-11 conceptualisation of AjD. Although the strong correlations between the two factors might suggest that AjD could be considered a unidimensional construct for research purposes, the ICD-11 distinction remains essential for clinical diagnosis. Internal consistency was high, with McDonald’s ω indicating good reliability. These findings reinforced the factorial validity of the IADQ across different national populations.

Beyond dimensionality and reliability, it is also important to determine whether the IADQ measures AjD equivalently across demographic groups. The observed measurement invariance across gender and language suggests that the IADQ assesses the construct in broadly comparable ways across these groups. Given the absence of significant mean score differences within these groups, the results likely reflect true equivalence rather than measurement bias. This has important implications for practical use, as participant responses – at least in comparable populations – can be interpreted as reflecting their personal experience, rather than demographic influences.

The IADQ demonstrated configural invariance across diagnostic status, indicating that the underlying factor structure was comparable between groups. However, metric invariance was not supported, suggesting that item-factor relationships differed as a function of diagnostic status. This finding implies that, although the construct is conceptualised similarly across groups, individual items may carry different psychological meaning or weight for participants with and without a diagnosis of AjD. Consequently, comparisons of latent relationships and latent mean scores across diagnostic groups are not supported and should be interpreted with caution. One explanatory hypothesis to explore in future research concerns clinical versus subclinical processing, whereby individuals who do not meet diagnostic criteria may interpret items in a more reflective or situational manner, whereas those who do may respond based on more chronic or pervasive experiences.

The IADQ demonstrated useful concurrent validity, showing strong correlations with measures of depression and anxiety and a more moderate correlation with PTSD. This pattern is consistent with prior research,^14,16,28,30^ indicating that while AjD shares features with other affective and stress-related disorders, it remains a distinct clinical construct. The comparatively weaker association with PTSD suggests that AjD may be more closely related to transient, stressor-driven maladaptive responses rather than the prolonged and intrusive symptomatology characteristic of PTSD, indicating some degree of discriminative capability.^30^ However, recent concerns regarding the validity of the 0–5 scoring system of the PC-PTSD-5 caution against overinterpretation.^62,63^ The observed correlation (*r* = 0.325) may further reflect shared measurement artefacts, such as English language comprehension difficulties.

### Risk and protective factors

Beyond establishing measurement rigour, examining factors associated with vulnerability and protection offered further insight into the practical relevance of AjD in occupational contexts. In this sample of educated employed adults, neither gender nor home language significantly affected IADQ completion or outcome, suggesting that AjD affected demographically diverse workers in this sample similarly. Although a small age difference was observed between those diagnosed with and without AjD, the findings suggested minimal age-related vulnerability in this sample.

Psychological resilience emerged as a significant but modest protective factor, with higher CD-RISC-10 scores reducing the likelihood of AjD diagnosis. Literature on the intersection of psychological resilience and AjD is sparse, and further research is encouraged to explore whether enhancing personal resilience, for example, through the development of adaptive coping strategies, may mitigate the occurrence of AjD.^64^

Previously, ACE have been recognised as a risk factor for poor long-term mental health outcomes, including AjD,^65^ across national, cultural and economic groups. South Africa has many young adults with a history of ACE,^66^ and the presence of AjD could serve as an indicator of such experiences, facilitating referrals for appropriate intervention. Further research is required to more deeply understand the intersection of ACE and AjD within the SA context.

### Clinical utility

Beyond psychometric performance, an important question is whether the IADQ functions effectively as a screening tool in applied occupational health settings. From a practical perspective, the IADQ diagnostic algorithm demonstrated high accuracy in this employed workplace sample, supporting its potential usefulness for clinical screening in similar educated occupational populations. However, it is important to caution that screening is not diagnosis. Despite the IADQ’s demonstrated accuracy, it cannot substitute careful clinical assessment to determine diagnosis and treatment. The IADQ is essentially a screening tool, not a psychometric replacement for clinical diagnosis. In this regard, the grey zone approach may offer a nuanced framework in settings where the inclusion of symptom severity could enhance decision-making and triage in clinical practice, particularly in resource-restricted environments. In this environment, it may offer two benefits: Firstly, the identification of a lower threshold for closer monitoring and an upper threshold for urgent intervention could offer a practical solution to the challenge of binary diagnostic cut-offs. Secondly, the grey zone offers a response to contextual constraints, as it allows for a graduated triage towards further assessment. This may allow for the early identification of individuals at risk for prolonged distress while preventing unnecessary referrals for transient stress reactions. The thresholds used here should be considered illustrative, and future research is required to refine these thresholds across diverse populations for broader applicability.

The IADQ specifies that symptom onset *usually* occurs within 1 month of the identified stressor, and participants’ responses to item 16 (indicating onset within 1 month) were incorporated into the diagnostic algorithm. However, clinical interview records indicated that many participants expressed uncertainty regarding the exact timing of symptom onset, raising concerns about the accuracy of retrospective recall. In such cases, clinicians may need to apply the 1-month criterion with discretion, in line with World Health Organization (WHO) guidance that recognises longer delays between exposure to a stressor and the emergence of clinically significant symptoms that impair functioning.^67^ The use of symptom severity thresholds may further enhance clinical decision-making, particularly in cases where timing of onset is uncertain but symptom severity is high.

In the context of occupational health, the findings underscore the importance of addressing mental health in workplace settings.^22^ Given that AjD is often associated with prolonged sickness absence and substantial costs related to lost productivity,^23^ the ability of the IADQ to identify at-risk individuals is relevant to occupational health services. Practically, the IADQ can be incorporated into existing occupational health monitoring protocols, and used as screening tool to guide referral for further assessment. Once identified, AjD, linked to work and non-work-related stress, can be mitigated by supportive workplace interventions, such as early intervention, resilience training and employee assistance programmes.^68,69^

### Language

Comparable prevalence estimates and demonstrated measurement invariance of the English-version IADQ across language groups provide preliminary support for its clinical utility in an educated, skilled worker sample. Nevertheless, several ethical and practical concerns remain. Persistent language barriers in clinical settings risk further marginalising English-illiterate and less-educated SAs,^70,71^ emphasising the ongoing need for better language competencies among healthcare providers,^42^ and for the validation of IADQ translations into other SA languages.^72,73^ Evidence from WEIRD countries supports the feasibility of translation.^14,28,29,30^

### Limitations

Our study has several limitations. Firstly, it used a convenience sample which was not nationally representative, with an over-representation of men and individuals with higher educational attainment. Consequently, the findings may not generalise beyond similarly educated, employed populations, and caution is warranted when extrapolating results to broader community, clinical or psychiatric samples. Secondly, diagnostic exclusions specified in the ICD-11 (e.g. uncomplicated bereavement, prolonged grief disorder or acute stress reaction) could not be applied because these variables were not available in the archived dataset. As a result, adjustment of AjD prevalence was not possible, raising the possibility of an overestimation of diagnostic rates. Thirdly, no study could be located to compare the high diagnostic accuracy findings, and these findings remain under-interpreted. In addition, the IADQ and the clinical interview were administered within a short time frame (within 24 h of each other), increasing the risk of contamination between assessments. Associations may therefore have been inflated by common method variance, reflecting carryover and consistency effects arising from the temporal proximity of measurement.^74^ Fourthly, the clinical interview used as the reference standard was conducted as part of routine care and did not include independent diagnostic verification or inter-rater reliability assessment. Fifthly, as discussed, all instruments were administered in English within a multilingual population, which may have influenced comprehension and response patterns.

### Future directions

The findings indicated that the IADQ is suitable for research applications and highlight several promising avenues for future investigation. Firstly, the intersection of personal resilience and AjD, as well as the relationship between ACE and AjD, warrant further examination. Secondly, research on the role of personality traits, particularly maladaptive traits, as potential risk factors for AjD may yield valuable insights into the disorder’s aetiology and inform prevention efforts.^64^

From a psychometric screening perspective, additional validation efforts could enhance accessibility to mental healthcare. These include translating the instrument into additional SA languages, conducting corresponding psychometric validation studies and prospectively refining grey zone thresholds across diverse populations to improve broader applicability.

## Conclusion

The IADQ demonstrated good psychometric properties and promising clinical utility for workplace mental health screening within this SA occupational sample. The grey zone approach may provide a practical framework for identifying individuals at varying levels of risk who may benefit from monitoring or early intervention in resource-constrained settings.
